# Molecular characterization and SNP identification using genotyping-by-sequencing in high-yielding mutants of proso millet

**DOI:** 10.3389/fpls.2023.1108203

**Published:** 2023-05-18

**Authors:** Neethu Francis, Ravikesavan Rajasekaran, Veera Ranjani Rajagopalan, S. Vinothini Bakya, Raveendran Muthurajan, Ashwini Girish Kumar, Senthil Alagarswamy, Iyanar Krishnamoorthy, Chitdeshwari Thiyagarajan

**Affiliations:** ^1^ Department of Genetics and Plant Breeding, Centre for Plant Breeding and Genetics, Tamil Nadu Agricultural University, Coimbatore, India; ^2^ Centre for Plant Breeding and Genetics, Tamil Nadu Agricultural University, Coimbatore, India; ^3^ Department of Plant Biotechnology, Centre for Plant Molecular Biology and Biotechnology, Tamil Nadu Agricultural University, Coimbatore, India; ^4^ Directorate of Research, Tamil Nadu Agricultural University, Coimbatore, India; ^5^ Parse Biosciences, Stockholm, Sweden; ^6^ Department of Crop Physiology, Tamil Nadu Agricultural University, Coimbatore, India; ^7^ Department of Millets, Centre for Plant Breeding and Genetics, Tamil Nadu Agricultural University, Coimbatore, India; ^8^ Department of Soil Science and Agricultural Chemistry, Tamil Nadu Agricultural University, Coimbatore, India

**Keywords:** proso millet, molecular, high yield, genotype-by-sequencing, SNP, mutants

## Abstract

Proso millet (*Panicummiliaceum* L.) is a short-duration C_4_ crop that is drought tolerant and nutritionally rich and can grow well in marginal lands. Though the crop has many climate-resilient traits like tolerance to drought and heat, its yield is lower than that of common cereals like rice, wheat, and maize. Being an underutilized crop, the molecular resources in the crop are limited. The main aim of the present study was to develop and characterize contrasting mutants for yield and generate functional genomic information for the trait in proso millet. Gamma irradiation-induced mutant population was screened to identify high-yielding mutants, which were evaluated up to M_4_ generation. One mutant with a dense panicle and high yield (ATL_hy) and one with a lax panicle and low yield (ATL_ly) along with the wild type were sequenced using the genotyping-by-sequencing approach. The variants detected as single nucleotide polymorphisms (SNPs) and insertions–deletions (InDels) were annotated against the reference genome of proso millet. Bioinformatic analyses using the National Center for Biotechnology Information (NCBI) and UniProt databases were performed to elucidate genetic information related to the SNP variations. A total of 25,901, 30,335, and 31,488 SNPs, respectively, were detected in the wild type, ATL_hy mutants, and ATL_ly mutants. The total number of functional SNPs identified in high-yielding and low-yielding mutants was 84 and 171, respectively. Two functional SNPs in the high-yielding mutant (ATL_*hy*) and one in the low-yielding mutant (ATL_*ly*) corresponded to the gene coding for “E3 ubiquitin-protein ligase UPL7”. Pathway mapping of the functional SNPs identified that two SNPs in ATL_ly were involved in the starch biosynthetic pathway coding for the starch synthase enzyme. This information can be further used in identifying genes responsible for various metabolic processes in proso millet and in designing useful genetic markers.

## 1 Introduction

Proso millet (*Panicum miliaceum* L.) (2n = 4x = 36) is a short-duration, self-pollinated C_4_ crop that can grow on marginal lands with minimal water and nutrients. It is rich in protein, fiber, vitamins, and minerals and has a very low glycemic index. Globally, the crop is known by different names like broom corn millet, common millet, *hog* millet, French white, and *hersey* (Rajput et al., 2014; Gomashe, 2017). It is one of the oldest domesticated crops and is often referred to as an ancient crop. China is a widely acknowledged region of domestication of the crop. However, some evidence suggests two independent centers of origin, i.e., China and Eastern Europe (Lu et al., 2005; Lu et al., 2009; Bettinger et al., 2010). The crop is distributed in almost all the continents of the world except for Antarctica, i.e., Asia, Australia, Africa, North America, South America, and Europe (Cavers and Kane, 2016; Vetriventhan et al., 2019). In India, it is mainly grown in Uttar Pradesh, Bihar, Madhya Pradesh, Maharashtra, Karnataka, Andhra Pradesh, and Tamil Nadu (TNAU 2021). Proso millet production in India as of 2017 estimates is 22,000 tonnes (AICRP, 2017).

Germplasm sources of the crop are maintained mainly in Russia, China, Ukraine, India, and the USA (Upadhyaya et al., 2016). *Panicum capillare* and *Panicum repens* are weedy wild relatives of *P. miliaceum*. In *P. miliaceum*, subsp. *miliaceum* has cultivated species, and subsp. *ruderale* has weedy genotypes (Cavers and Kane, 2016). The subsp. *miliaceum* is divided into five races based on its panicle morphology, i.e., Miliaceum, Patentissimum, Contractum, Compactum, and Ovatum (Rajasekaran et al., 2023).

It is inherently drought-tolerant and the most water use-efficient cereal crop. These characteristic features make the crop ideal for the present climate change scenario particularly due to global warming and increased surface temperatures. However, one of the major limitations of the crop is its lower yield when compared to major cereals. Hence, improving the crop’s production and productivity is essential to advocate its large-scale commercial cultivation.

Proso millet is an underutilized crop, and genomic resources present in the crop are limited. Pioneer studies utilized random-amplified polymorphic DNA (RAPD), amplified fragment length polymorphism (AFLP), inter simple sequence repeat (ISSR), and simple sequence repeat (SSR) markers in generating molecular markers and genomic information. One of the first studies that used next-generation sequencing (NGS) platforms made use of the genotyping-by-sequencing (GBS) approach to map quantitative trait loci (QTLs) and construct a linkage map for the crop (Rajput et al., 2016). However, recently, the nuclear and chloroplast genomes of the crop have been sequenced (Yue et al., 2016b; Cao et al., 2017; Nie et al., 2018; Shi et al., 2019; Zou et al., 2019). A genome-wide association study detected marker–trait associations for seed-related traits (Boukail et al., 2021). Apart from this, few transcriptomic approaches have given insights into the stress tolerance mechanisms of the crop (Yue et al., 2016a; Yue et al., 2016b; Zhang et al., 2019). In spite of this, the availability of gene-specific molecular tools and functional information is scarce.

The main aim of the present study was to develop and characterize contrasting mutants for yield and generate functional genomic information for the trait in proso millet. Molecular characterization of the mutants ATL_*hy*, ATL_*ly*, and wild was carried out to detect the single-nucleotide polymorphisms (SNPs) and insertions–deletions (InDels) induced through mutagenesis, understand their type and chromosome distribution, and delineate the functional SNPs and their role in biological pathways. Considering the polyploid nature of the crop, the polygenic nature of targeted traits, scarce molecular resources and funding availability, the GBS approach was relied upon for characterization. GBS is a rapid, cost-effective, and informative sequencing approach, particularly in crops with large genome sizes.

## 2 Materials and methods

### 2.1 Development and identification of high-yielding mutants

Seeds of the proso millet variety ATL 1, obtained from the Centre of Excellence in Millets, Tamil Nadu Agricultural University, Athiyanthal, Tamil Nadu, India, were irradiated with gamma mutagen to develop the mutant population. Irradiation of the seed material was carried out at Bhabha Atomic Research Centre (BARC), Mumbai, India. For each dose, 1,500 dry seeds were irradiated using a gamma chamber having a cobalt-60 source. A pilot study was conducted with 10 doses of gamma, i.e., 100 to 1,000 Gy, to determine the optimum treatment doses of gamma mutagen in the crop. Treatment doses 400 Gy followed by 500 Gy were found to be the best treatment doses based on probit analysis, mutation frequency, mutagenic effectiveness, and efficiency percentages. The experiment was carried out at the Department of Millets, Tamil Nadu Agricultural University, Coimbatore, India. Seeds from M_1_ plants were forwarded to M_2_ generation on an ear-to-row basis to constitute 200 M_2_ families. Seeds were sown in rows on 3-m-long ridges and maintained at a spacing of 20 cm between plants post-thinning. Screening for yield-contributing traits like flag leaf length (FLL), flag leaf breadth (FLB), panicle length (PL), number of tillers (NT), number of panicles (NP), plant height (PH), plant vigor, and days to flowering (DF) was carried out in M_2_ and M_3_ generations. Eighteen putative high-yielding mutants were forwarded to M_3_ as ear-to-row progenies and evaluated for yield-contributing traits. Thirteen high-yielding families were raised in M_4_ with two rows per family in a randomized blocks design (RBD) with three replications, and eight high-yielding mutant families were confirmed after evaluation (Francis et al., 2022). Additionally, mutants with low yield and yield-contributing characters and contrasting panicle attributes were also forwarded and evaluated till M_4_. Two representative contrasting mutants for yield and panicle density, *viz*., ATL1 500-45-3 (represented as ATL_*hy*) and ATL1 400-43-1 (represented as ATL_*ly*), were identified for sequencing and molecular characterization. The description of yield mutants sequenced and characterized is given in **Table 1**.

**Table 1:** Phenotypic description and biometric observation of wild type and mutants.

Characters	Wild	ATL_*hy*	ATL_*ly*	Wild	ATL_*hy*	ATL_*ly*
	M_3_ generation			M_4_ generation		
**Panicle type**	Semi compact	Dense, arched	Lax, spreading	Semi compact	Dense, arched	Lax, spreading
**Yielding nature**	Moderate	High	Low	Moderate	High	Low
**PH (cm)**	129.55	165.00	118.00	98.91	132.41	95.20
**FLL (cm)**	29.87	39.00	32.50	27.51	32.33	29.50
**FLB (cm)**	2.05	2.60	1.60	1.80	2.15	1.70
**PL (cm)**	40.02	48.00	35.00	37.73	46.15	40.50
**NP**	9.40	23.00	16.00	12.00	17.60	15.00
**NT**	7.30	20.00	10.00	6.50	11.50	6.00
**DF**	42.60	44.00	38.00	38.80	44.90	37.00
**DM**	75.40	79.00	73.00	72.80	77.80	72.00
**FY (g)**	63.35	68.90	41.4	30.40	61.61	28.90
**GY (g)**	23.95	73.92	18.5	15.20	34.23	11.93

PH, plant height; FLL, flag leaf length; FLB, flag leaf breadth; PL, panicle length; NP, number of panicles; NT, number of tillers; DF, days to flowering. DM, Days to maturity; FY, Fodder yield; GY, Grain yield.

### 2.2 Plant DNA extraction and quality control

The DNA was extracted from young fresh leaves following the modified cetyltrimethylammonium bromide (cTAB) method (Murray and Thompson, 1980; Murray et al., 2008). NanoDrop^®^ 2000 spectrophotometer was used to quantify the DNA purity by assessing the OD_260_/OD_280_ ratio. Sample DNA, with an OD_260_/OD_280_ ratio of 1.8 to 2.0 and a total amount of more than 1.5 μg, was qualified for library construction.

### 2.3 Library preparation and high-throughput DNA sequencing

**Figure 1:**
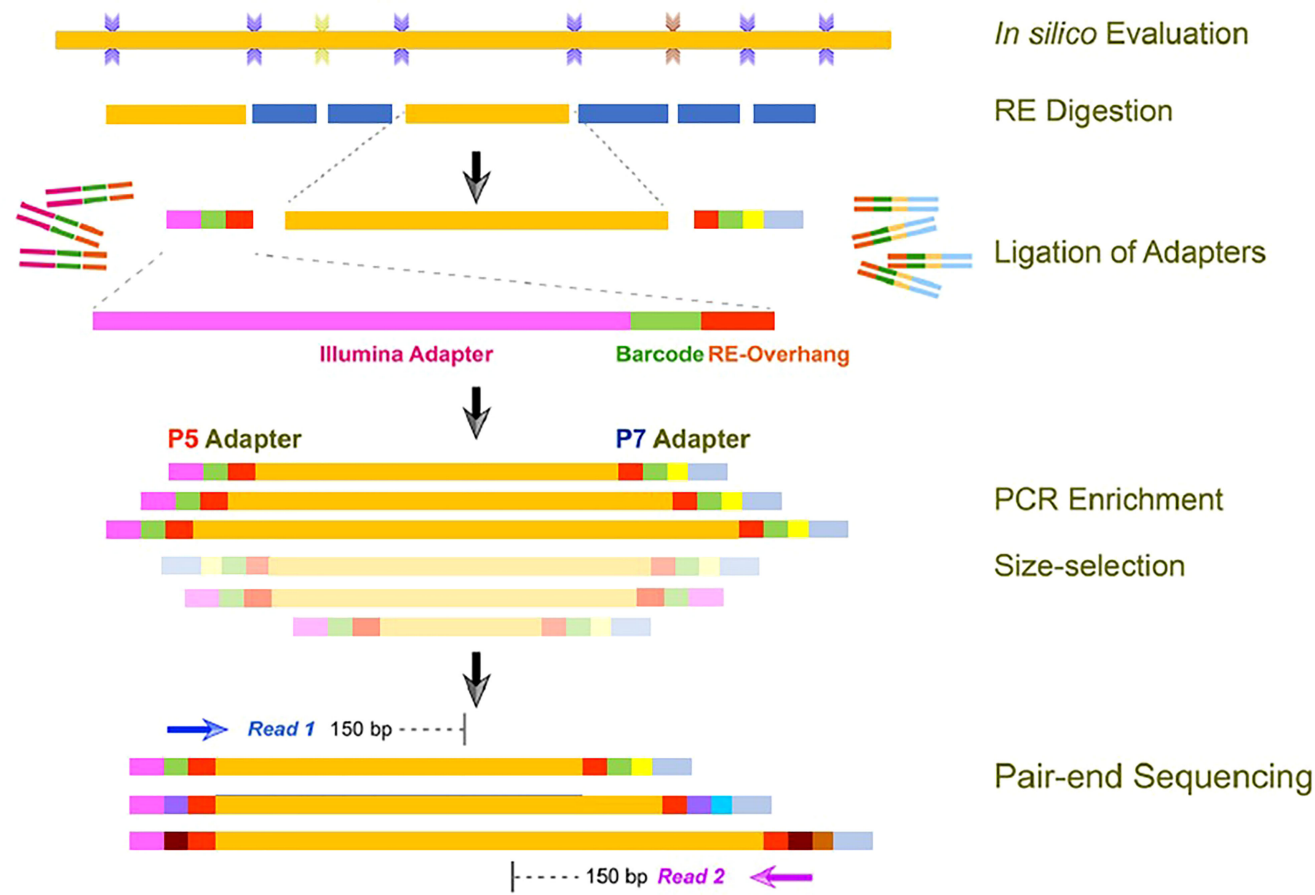
Experimental procedures of library preparation for high-throughput sequencing.

High-throughput sequencing was performed at Oneomics Private Limited (India) by using a modified in-house pipeline based on a protocol adapted from Elshire et al. (2011). The basic outline of the experimental procedures carried out for library preparation is depicted in **Figure 1**. Based on the *in silico* digestion analysis of the proso millet genome assembly, the restriction enzyme and fragment sizes were determined. Further, the genomic DNA (0.3~0.6 μg) was digested completely using the type II restriction endonuclease (*Mse*I). The digested fragments were ligated with two barcoded adapters either with a compatible sticky end or with the primary digestion enzyme and the Illumina P5 or P7 universal sequence. Tags containing adapters were amplified through PCR, DNA fragments of different samples were pooled, and desired fragments were recovered. The concentration and insert size of the prepared library were analyzed using Qubit^®^ 2.0 fluorometer and Agilent^®^ 2100 bioanalyzer, respectively. Libraries with appropriate insert size and concentration greater than 2 nM were qualified for high-throughput sequencing. High-throughput DNA sequencing was then performed on the Illumina platform, with a read length of 144 bp at each end.

### 2.4 Bioinformatic analysis of sequenced data

#### 2.4.1 Raw data

The original image data obtained from high-throughput sequencers were transformed to raw data (raw reads) by base calling using CASAVA software version 1.8. The sequences and corresponding sequencing quality information were stored in a FASTQ file.

#### 2.4.2 Quality control of sequence data

The sequencing error rate distribution was examined over the full length of the sequence to detect any sites (base positions) with an unusually high error rate, where incorrect bases may be incorporated at abnormally high levels. Bases with sequencing error rates below 1% were chosen. The quality distribution check was performed to ensure the quality of downstream analyses. The sequencing quality for the majority of bases is required to be greater than Q20. As a normal feature of sequencing, base quality is usually lower at the end of a sequence than that at the beginning.

Raw data obtained from sequencing contain adapter contamination and low-quality reads. These sequencing artifacts may increase the complexity of downstream analyses, and therefore, quality control steps were utilized to remove them. Consequently, all the downstream analyses are based on clean reads. The quality control steps are as follows: 1) discard the paired reads when either read contains adapter contamination, 2) discard the paired reads when uncertain nucleotides (N) constitute more than 10% of either read, and 3) discard the paired reads when low-quality nucleotides (base quality less than 5) constitute more than 50% of either read.

#### 2.4.3 Mapping clean reads to reference genome

The mapping rates of samples reflect the similarity between each sample and the reference genome. The reference genome used for mapping was the GeneBank assembly accession “GCA_003046395.2” submitted by the Shanghai Center for Plant Stress Biology (Zou et al., 2019). The depth and coverage are indicators of the evenness and homology of the reference genome. The effective sequencing data were aligned with the reference sequence through the Burrows–Wheeler Alignment (BWA) software, and bam result files were generated (Li and Durbin, 2009). The number of clean reads mapped to the reference assembly, including both single-end reads and reads in pairs, is given as “Mapped Reads”. The total number of effective reads in clean data is given as “Total Reads”. The number of the enzyme cutting fragments present is given as “Tag number”. The ratio of the reference genome mapped reads to the total sequenced clean reads is called the “Mapping Rate”. The average depth of mapped reads at each site, calculated by the total number of bases in the mapped reads divided by the size of the assembled genome, is expressed as “Average depth”. The percentage of the assembled genome with more than one read at each site is mentioned as “Coverage at least 1X”. The percentage of the assembled genome with more than four reads’ coverage at each site is designated as “Coverage at least 4X”.

#### 2.4.4 Variant calling and annotation

The perfectly matched and aligned sequences from BWA were processed further for SNP calling through the SAMtools mpileup tool (Li and Durbin, 2009). The description of SNPs and InDels detected are provided as **Supplementary Material**. SNP calls were employed using the following criteria: i) minimum read depth of 6 and ii) mapping quality <40. ANNOVAR was used to perform the annotation of SNPs and InDels and also to identify synonymous and non-synonymous SNPs (Wang et al., 2010).

#### 2.4.5 Identification of functional SNPs and pathway mapping

The SNPs between the wild type and mutants were identified by filtering the annotated data. Mutation-induced non-synonymous, stop gain, and stop loss SNPs were identified by comparing wild-type and mutant alleles. Bioinformatic analyses using the National Center for Biotechnology Information (NCBI) and UniProt databases were performed for pathway mapping of functional SNPs.

## 3 Results

**Figure 2:**
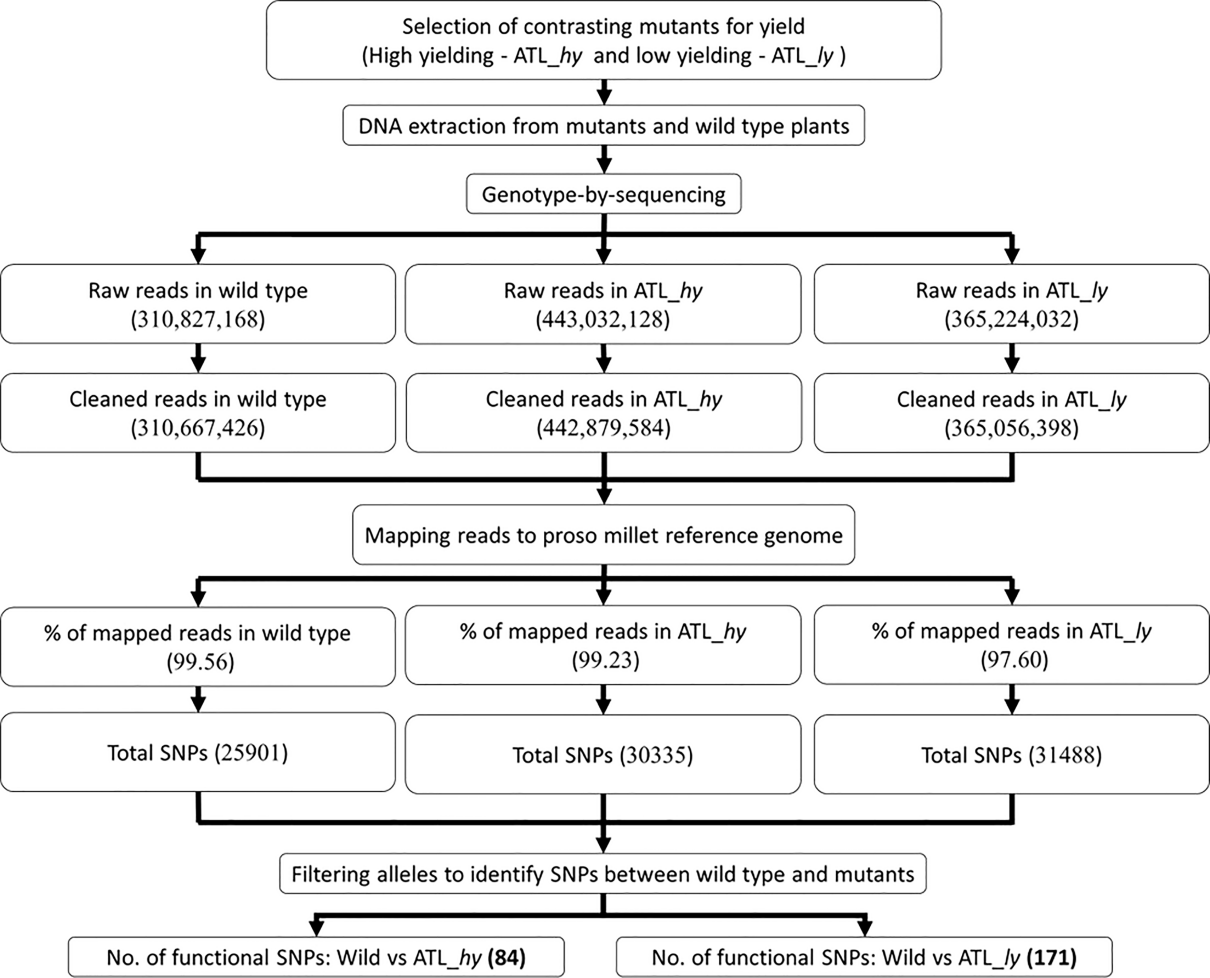
Flowchart of GBS-based molecular characterization of wild type and mutants. GBS, genotyping-by-sequencing.

The summary of the methods followed for molecular characterization and mapping of mutants is represented as a flowchart in **Figure 2**. The summary of clean data and mapping statistics after sequence alignment of wild type and mutants to the reference genome is given in **Table 2**. The error rate was 0.03% in all the samples, and Guanine-Cytosine (GC) content ranged from 40.95 (wild) to 41.06 (ATL_*ly*). A total of 99.56% reads from wild type, 99.23% from ATL_*hy*, and 97.60% reads from low mutant were mapped onto the reference genome. The average depth of sequencing or the average number of reads at a particular location ranged from 5.25 (wild type) to 7.29 (ATL_*hy*).

**Table 2:** GBS data summary and mapping statistics.

	Wild type	ATL_*hy*	ATL_*ly*
**Raw bases**	310,827,168	443,032,128	365,224,032
**Cleaned bases**	310,667,426	442,879,584	365,056,398
**Error rate (%)**	0.03	0.03	0.03
**GC content**	40.95	40.52	41.06
**Total reads**	2,026,260	2,869,588	2,323,956
**Mapped reads**	2,017,248	2,847,587	2,268,246
**Mapping rate (%)**	99.56	99.23	97.60
**Average depth (X)**	5.25	7.29	5.82
**Tag number**	451,159	456,535	461,472
**Coverage 1× (%)**	6.58	6.68	6.68
**Coverage 4× (%)**	2.86	3.36	3.03

GBS, genotyping-by-sequencing.

Mapping of reads identified variations from the reference genomes as SNPs and InDels, a summary of which is given in **Table 3**. A total of 25,901, 30,335, and 31,488 SNPs, respectively, were detected in the wild type, ATL_*hy* mutants, and ATL_*ly* mutants. Intergenic SNPs were higher in number compared to the genic SNPs in all the samples. Among the genic SNPs, intronic SNPs were more than the exonic SNPs. Among the exonic SNPs, non-synonymous SNPs were the predominant type followed by the synonymous SNPs, and stop loss SNPs were the rarest among both mutants and wild type. Transitions were higher than transversions among all the genotypes. A total of 1,977 indels in wild type, 2,499 indels in high yielding mutant, and 2,461 InDels in low yielding mutant were detected. Similar to the trend in SNPs, the intergenic InDels were higher than the genic InDels. Likewise, intronic InDels were higher than exonic InDels. In the exonic region, frameshift deletions were more common than non-frameshift deletions. Stop gain InDels were very rare, while no stop loss InDels were detected. The overall number of deletions was higher than insertions in the mutants, while the number of insertions was higher in the wild type.

**Table 3:** Summary of GBS sequence data alignment and mapping of wild type and mutants against reference genome.

	Wild type	ATL_*hy*	ATL_*ly*
SNPs			
Upstream	1,292	1,613	1,554
Exonic: stop gain	18	24	17
Exonic: stop loss	2	2	3
Exonic: synonymous	243	272	293
Exonic: non-synonymous	492	538	582
Intronic	2,423	2,877	2,927
Splicing	9	8	14
Downstream	1,148	1,410	1,462
upstream/downstream	130	139	146
Intergenic	19,781	22,976	24,006
Ts	18,224	21,162	21,991
Tv	7,677	9,173	9,497
ts/tv	2.373	2.306	2.315
Total	25,901	30,335	31,488
InDels			
Upstream	181	233	228
Exonic: stop gain	1	0	0
Exonic: stop loss	0	0	0
Exonic: frameshift deletion	15	13	16
Exonic: frameshift insertion	7	7	10
Exonic: non-frameshift deletion	2	4	4
Exonic: non-frameshift insertion	3	5	5
Intronic	298	371	341
Splicing	1	1	1
Downstream	148	209	206
Upstream/downstream	18	24	30
Intergenic	1,261	1,580	1,567
Insertion	995	1,241	1,222
Deletion	982	1,258	1,239
Total	1,977	2,499	2,461

GBS, genotyping-by-sequencing; SNPs, single-nucleotide polymorphisms.

A comparison of alleles between the wild type and mutants identified the SNPs induced through mutagenesis in the mutants, and the summary is presented in **Table 4**. The molecular characterization of two contrasting mutants and control plants revealed insights into the type of mutations induced in the genotypes. A total of 10,198 SNPs in the high-yielding mutant and 18,163 SNPs in the low-yielding mutant were detected in comparison with the wild type. The intergenic, intronic, and exonic SNPs were higher in ATL_*ly* compared to ATL_*hy*. Among the exonic SNPs, non-synonymous polymorphism was more compared to synonymous, stop gain, and stop loss SNPs. The number of homozygous SNPs was more than 50% in the low-yielding mutant than in the high-yielding mutant. Significant amino acid changes and protein changes will be contributed by the non-synonymous, stop gain, and stop loss SNPs (functional SNPs) present in the exonic region. The total number of functional SNPs was 84 and 171 for ATL_*hy* and ATL_*ly*, respectively. In wild type versus high mutant comparison, 81 non-synonymous, 2 stop gain, and 1 stop loss SNPs were found, whereas in wild type *vs.* ATL_*ly* comparison, 164 non-synonymous, 5 stop gain, and 2 stop loss SNPs were detected.

**Table 4:** Details of SNPs detected in mutants against wild type.

SNPs	Wild vs. ATL_*hy*	Wild vs. ATL_*ly*
Upstream	481	803
Exonic: stop gain	22	17
Exonic: stop loss	2	2
Exonic: synonymous	178	201
Exonic: non-synonymous	360	447
Intronic	1,177	1,776
Splicing	9	13
Downstream	476	774
upstream/downstream	50	79
UTR 3	117	209
UTR 5	59	93
Intergenic	7,267	13,738
Homozygous	4,041	11,064
Functional SNPs	84	171
Non-synonymous	81	164
Stop gain	2	5
Stop loss	1	2
Heterozygous	3,582	3,835
Total	10,198	18,163

SNPs, single-nucleotide polymorphisms.

**Figure 3:**
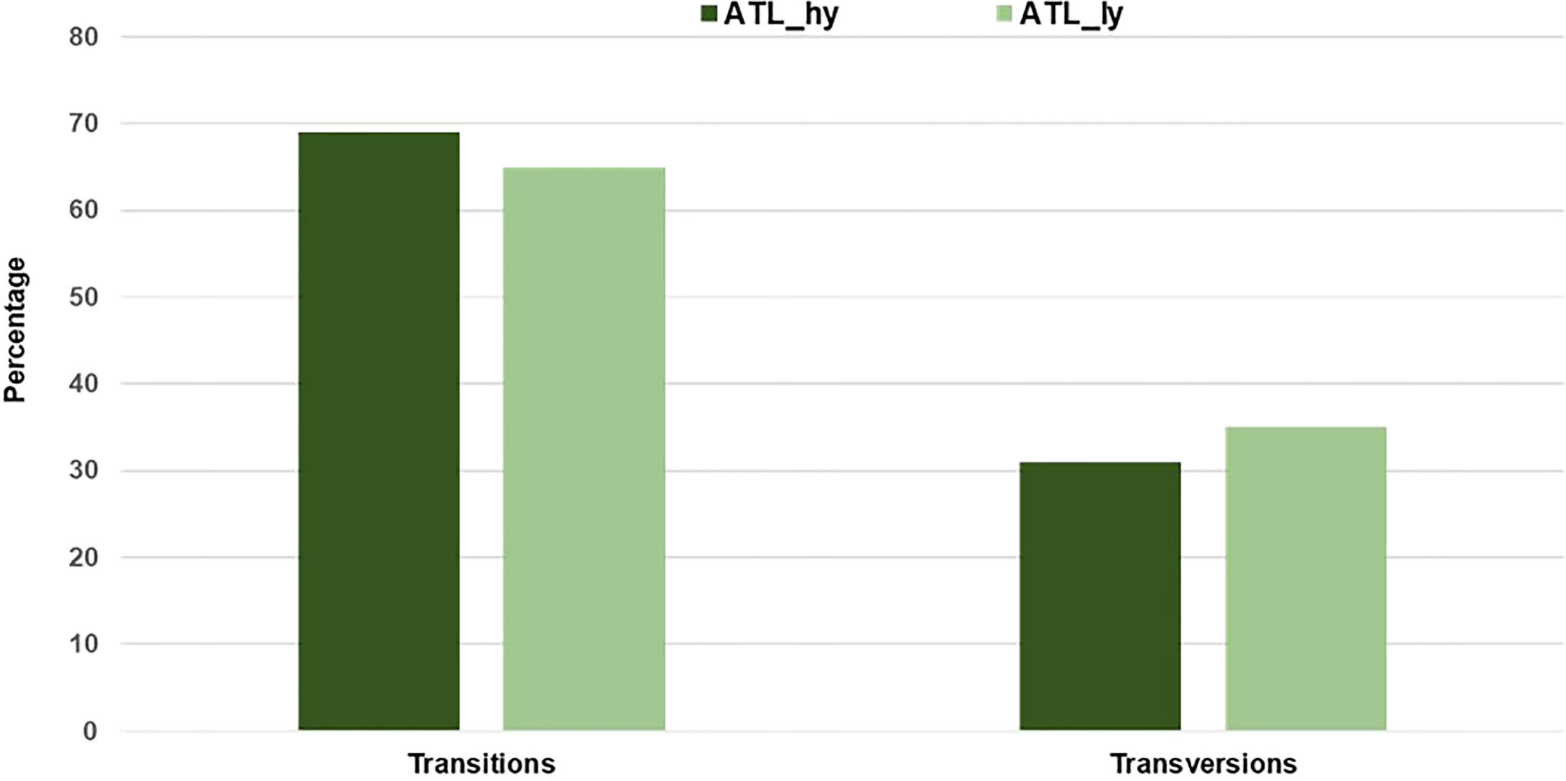
Percentage of transitions and transversions among the identified functional SNPs. SNPs, single-nucleotide polymorphisms.

**Figure 4:**
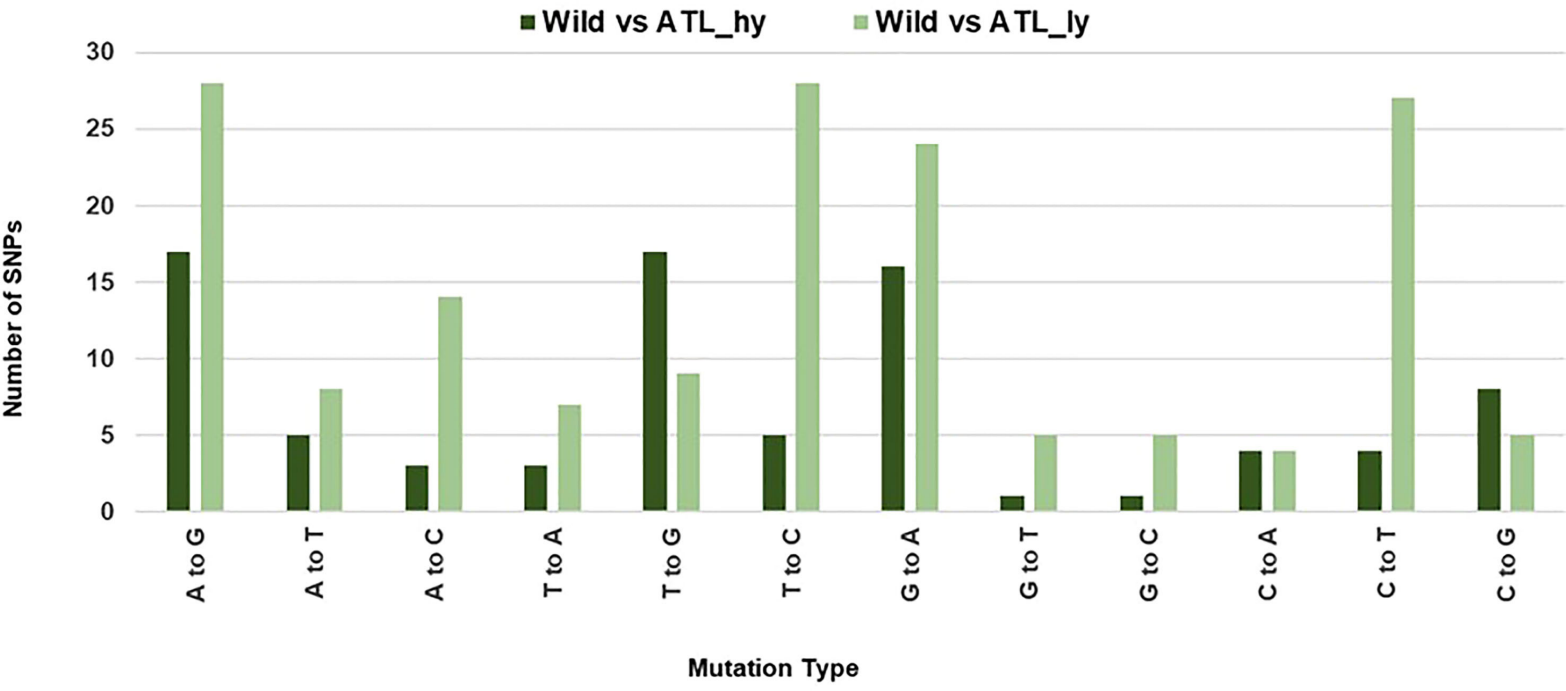
Mutation type distribution among the functional SNPs. SNPs, single-nucleotide polymorphisms.

**Figure 5:**
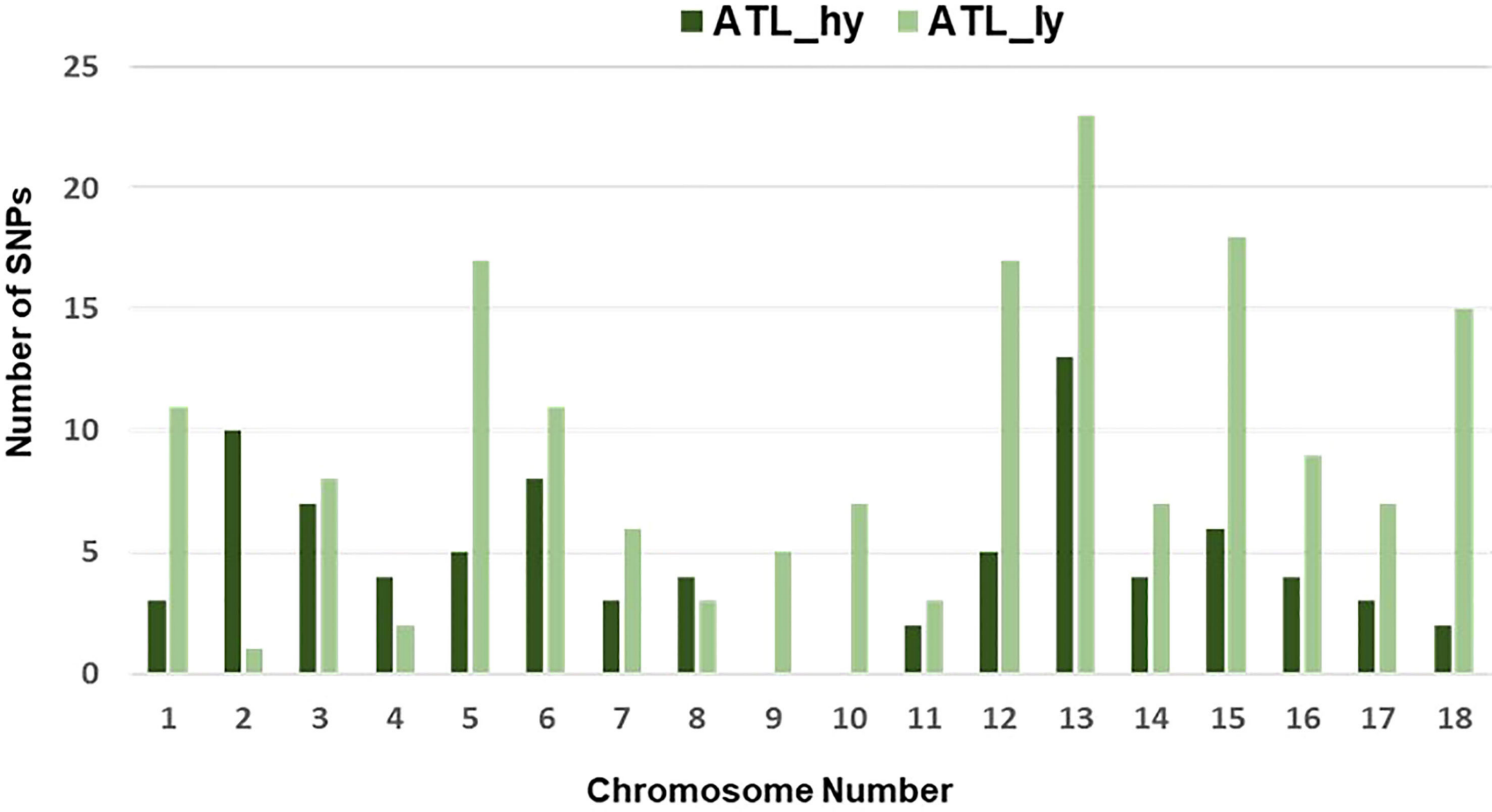
Chromosome-wise frequency distribution of functional SNPs. SNPs, single-nucleotide polymorphisms.

**Figure 6:**
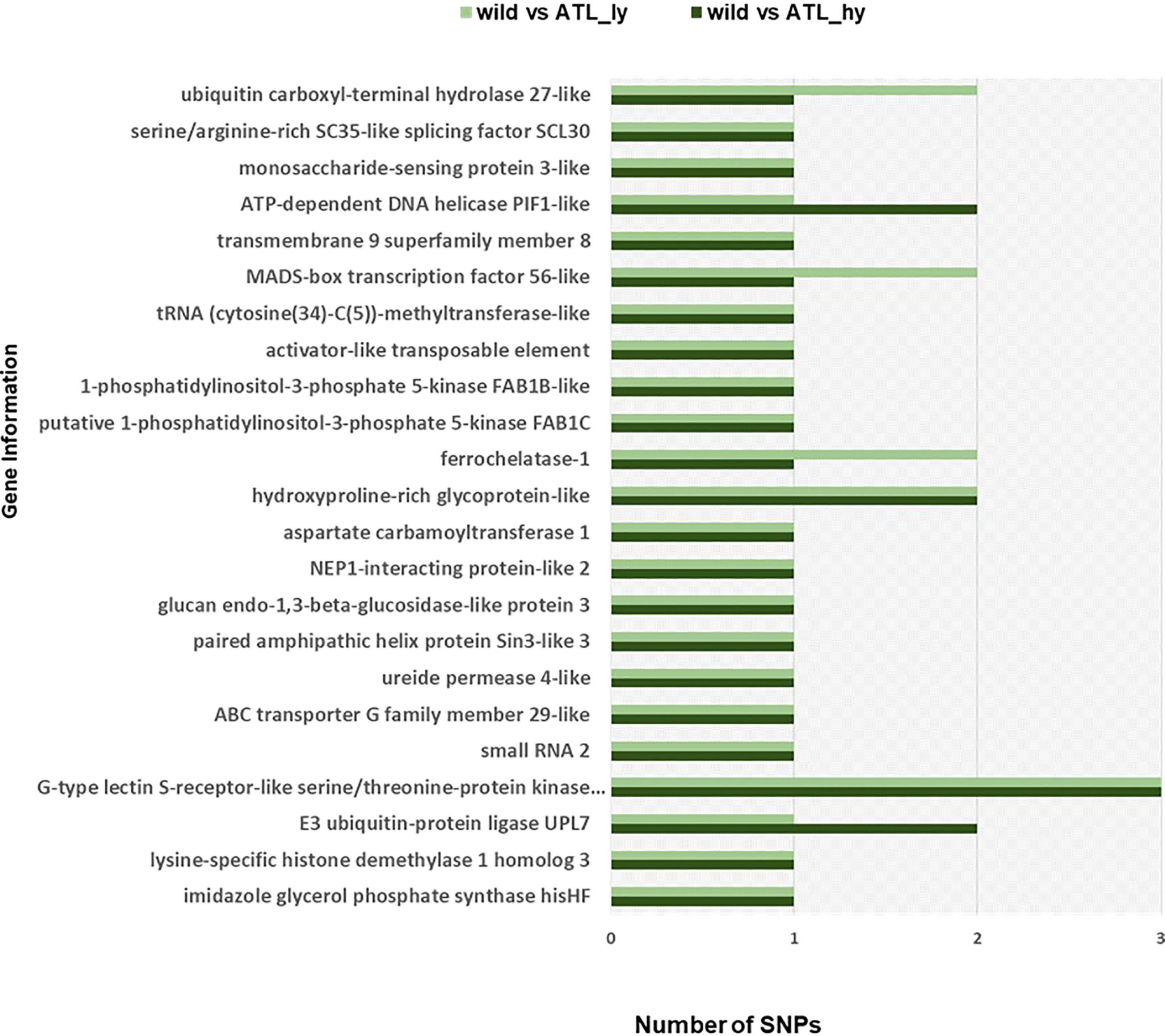
Gene information of functional SNPs overlapping among the mutants. SNPs, single-nucleotide polymorphisms.

The percentage distribution of transitions and transversions in mutants in comparison to the wild type is represented in **Figure 3**. For both mutants, transitions were higher than transversions. The number of transitions and transversions in the ATL_*hy* was 58 and 26, respectively. In the ATL_*ly*, the number of transitions and transversions was 111 and 60, respectively. Distribution of the different types of transition and transversion mutations that occurred as functional SNPs is depicted in **Figure 4**. These SNPs were distributed on 18 pseudochromosomes or linkage groups. The chromosome-wise frequency distribution of the identified functional SNPs in the mutants is given in **Figure 5**. The maximum number of SNPs for both mutants was identified on chromosome number 13. For high-yielding mutants, no SNPs were detected on chromosomes 9 and 10. The SNPs and corresponding gene information identified through bioinformatic analyses for the high and low-yielding mutants are given in the **Supplementary Material**. The gene information for the functional SNPs overlapping among the contrasting mutants is represented in **Figure 6**. Two functional SNPs that were detected only in the low-yielding mutant were present in a sequence coded for a protein involved in the starch biosynthesis pathway. These SNPs induced C-to-T transitions in the mutant.

## 4 Discussion

Mutation-induced genotypic changes can be due to one to many DNA level alterations in the form of InDels, translocations, and SNPs. Recently, there have been few sequencing-based studies to develop genetic and genomic resources in millets. The GBS approach was used to characterize the finger millet germplasm lines. A total of 23,000 SNPs were identified from the study across the genomes (Kumar et al., 2016). Next-generation sequencing of recombinant inbred lines (RILs) was performed to identify candidate genes controlling important morphological and agronomical traits in foxtail millet (Fukunaga et al., 2022). In proso millet, the first linkage map and QTLs for agronomic traits were reported by Rajput et al. (2016) using the NGS-based GBS platform. Another study generated 1,882 SNPs and developed genome-wide SNP markers by using the GBS approach among 190 accessions (Johnson et al., 2019). In a similar study, 85 diverse proso lines from 25 different countries were analyzed to identify SNPs and to study the diversity clustering of the accessions (Khound et al., 2022). However, the efforts in the crop are far behind the major crops like rice and wheat.

In this study, the GBS analysis of contrasting mutants for yield (ATL_*hy* and ATL_*ly*) and wild type revealed the type of mutations induced through mutagenesis. Wide genetic variation induced was detectable as SNPs and InDels. SNPs and InDels identified against reference genome were the highest in high-yielding mutants followed by low-yielding mutants and then in wild type. Compared to full genome sequencing, the GBS approach will have more missing data, and the detected total SNPs would be fewer than actually present. The full genome sequencing project of the crop reported 221,787 SNP markers and developed a genetic linkage map with 18 linkage groups (Zou et al., 2019). In the present study, intergenic SNPs were more in number, which is in accordance with the findings based on the mutations in the model plant *Arabidopsis*. Approximately 58% lower mutations were observed in the genic regions compared to the areas outside the gene in *Arabidopsis* (Monroe et al., 2022). Deletions were higher in the mutants compared to the wild type, which suggests that gamma rays induce more small deletions. Similar findings were reported in rice (Morita et al., 2009), and they found a higher proportion of small deletions in the mutants.

Comparison of the SNPs identified in wild type versus mutants showed that a greater number of functional SNPs were detected from the low-yielding mutant, which explains larger phenotypic variations in this mutant over the high-yielding mutant. Transitions were more common compared to transversions. The chromosome-wise spread of SNPs revealed that a major portion of the induced functional changes has occurred on chromosome 13. In high-yielding mutants, no functional SNPs were detected on chromosomes 9 and 10. The genes or proteins related to the SNPs identified narrow down the probable regions and genes responsible for the pleiotropic changes detected between the mutants. A genome-wide association study (GWAS) using SNP markers among the germplasm accessions of proso identified marker–trait correlations for 10 seed morphology and 3 agronomic traits (Boukail et al., 2021).

Further *in silico* analysis can identify specific SNPs involved in various metabolic pathways and processes modulating yield-attributing traits. Two functional SNPs in the high-yielding mutant (ATL_*hy*) and one in the low-yielding mutant (ATL_*ly*) corresponded to the gene coding for “E3 ubiquitin-protein ligase UPL7”. In rice, a loss-of-function mutant of E3 ubiquitin protein ligase (OsUPL2) called *large2* produced wide leaves, thick culms, large panicles, and increased grain number (Huang et al., 2021). Similarly, in *Brassica napus*, a ubiquitin protein ligase *BnUPL3* was reported, which regulated seed size and yield (Miller et al., 2019). SNPs in the “MADS-box transcription factor 56-like” gene were also detected in both ATL_*hy* and ATL_*ly*. In rice, MADS-box genes were found to be specially expressed in seed and panicle development (Arora et al., 2007).

Pathway mapping of the functional SNPs identified that two functional SNPs in ATL_ly were involved in the starch biosynthetic pathway coding for the starch synthase enzyme. Starch biosynthesis is well-studied in major cereals. It is an important factor that can influence crop yield. Though different enzymes contribute to the synthesis of starch, starch synthases are the major determinants of starch structure and amount in cereals (Irshad et al., 2021). Different classes of starch synthases are identified in plants (Liu et al., 2015). Transgenic introgression of rice soluble starch synthase I (heat tolerant) into wheat lines produced higher grain weight under stress in wheat (Tian et al., 2018).

Further validation of the identified SNP and confirmation using gene-targeted approaches can improve the understanding of the role of starch synthases in proso millet. This information can also be used in identifying genes responsible for various metabolic processes in proso millet and can be used in designing useful genetic markers. Unlike in common crops like rice, molecular information and molecular tools in proso millet are scanty, and these insights can aid in future genomics-assisted breeding programs in the crop. In a similar study in *Dendrobium*, the GBS-based SNPs were used for the characterization of mutants in the crop and the development of Kompetitive Allele Specific PCR (KASP) assay sets (Ryu et al., 2019). Similar attempts have been made in other crops like rapeseed and oil palm using GBS by Pootakham et al. (2015) and Ryu et al. (2021).

## Data availability statement

Original datasets used in the study are deposited in the Sequence Read Archive repository of NCBI, Bioproject accession number- PRJNA966134. This data can be found here: http://www.ncbi.nlm.nih.gov/bioproject/966134.

## Author contributions

NF- Conduct of research experiments, drafting of manuscript. RR, RM- Advise and supervision during conduct of experiment. VR - Bioinformatic analyses, Manuscript drafting. SB, AK- Bioinformatic analyses, manuscript drafting. SA, IK, CT- Planning of experiments. All authors contributed to the article and approved the submitted version.
