# Supplementary material for: Molecular characterization and SNP identification using genotyping-by-sequencing in high-yielding mutants of proso millet
Source: Front Plant Sci. 2023 May 18;14:1108203. doi: 10.3389/fpls.2023.1108203 (PMC10233037; doi:10.3389/fpls.2023.1108203)
Supplement: Supplementary file 1 [file DataSheet_1.docx]

Supplementary Material

**S 1. Classification of raw reads among the samples**

**
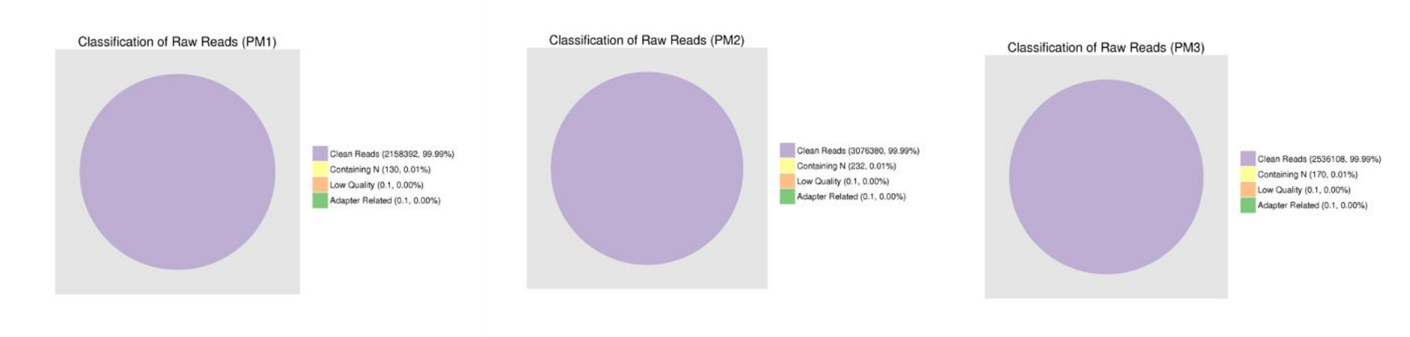
**

**S 2. Error rate distribution along the reads in the samples**

**
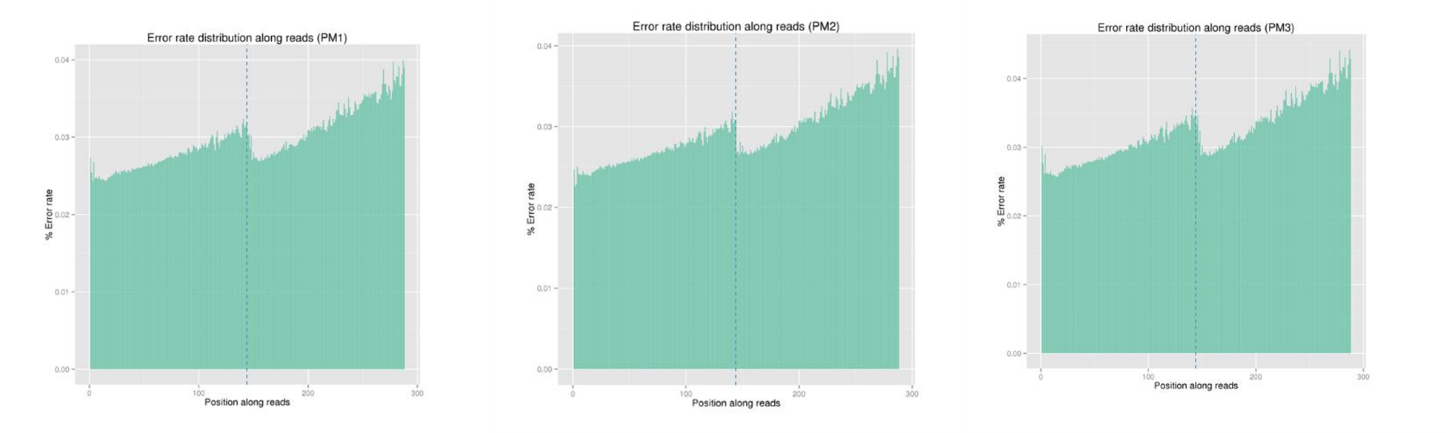
**

**S 3: Description of categories of SNPs and Indels detected**

| 1 | Upstream | SNPs/InDels located within 1 kb upstream (away from transcription start site) of the gene. |
| --- | --- | --- |
| 2 | Exonic SNPs | SNPs/Indels located in exonic region; Non-synonymous, Stop gain/loss and Synonymous |
|  | Non-synonymous | single nucleotide mutation with changing amino acid sequence |
|  | Stop gain/loss | a nonsynonymous SNP that leads to the introduction/removal of stop codon at the variant site |
|  | Synonymous | single nucleotide mutation without changing amino acid sequence |
| 3 | Exonic InDels | InDels located in exonic region; stop gain/loss, frameshift deletion/insertion and non-frameshift deletion/insertion |
|  | Stop gain/loss | InDel that leads to the introduction/removal of stop codon at the variant site |
|  | Frameshift Insertion/ deletion | InDel mutation changing the open reading frame with deletion or insertion |
|  | Non-frameshift insertion/deletion | InDel mutation without changing the open reading frame with deletion or insertion sequences of 3 or multiple of 3 bases |
| 4 | Intronic | SNPs/Indels located in intronic region; |
| 5 | Splicing | SNPs located in the splicing site (2 bp range of the intron/exon boundary). |
| 6 | Downstream | SNPs/Indels located within 1 kb downstream (away from transcription termination site) of the gene region. |
| 7 | Upstream/  Downstream | SNPs/Indels located within the < 2 kb intergenic region, which is in 1 kb downstream or upstream of the genes. |
| 8 | Intergenic | SNPs/Indels located within the > 2 kb intergenic region. |
| 9 | ts | Transitions, a point mutation that changes a purine nucleotide to another purine (A to G) or a pyrimidine nucleotide to another pyrimidine (C to T). Approximately two out of three SNPs are transitions. |
| 10 | tv | Transversions, the substitution of a (two ring) purine for a (one ring) pyrimidine or vice versa. |
| 11 | ts/tv | The ratio of transitions to transversions. |
| 12 | Total | The total number of SNPs. |

**S 4. Details of functional single nucleotide polymorphisms (SNPs) identified between wild type and high yielding mutant**

| **Sl. No.** | **Chromosome number** | **GenBank accession** | **Gene Locus** | **SNP type** | **Wild type** | **High yielding mutant** | **Gene Information** |
| --- | --- | --- | --- | --- | --- | --- | --- |
| 1 | 1 | CM009690.2 | C2845_PM01G05320 | nonsynonymous SNP | G G | A A | WEAK CHLOROPLAST MOVEMENT UNDER BLUE LIGHT 1-like isoform X3 |
| 2 | 1 | CM009690.2 | C2845_PM01G09740 | nonsynonymous SNP | A A | G G | imidazole glycerol phosphate synthase hisHF, chloroplastic |
| 3 | 1 | CM009690.2 | C2845_PM01G19760 | nonsynonymous SNP | T T | C C | lysine-specific histone demethylase 1 homolog 3 |
| 4 | 2 | CM009691.2 | C2845_PM02G02450 | nonsynonymous SNP | A A | G G | E3 ubiquitin-protein ligase UPL7 |
| 5 | 2 | CM009691.2 | C2845_PM02G02450 | nonsynonymous SNP | G G | A A | E3 ubiquitin-protein ligase UPL7 |
| 6 | 2 | CM009691.2 | C2845_PM02G11830 | nonsynonymous SNP | G G | A A | polyubiquitin-like |
| 7 | 2 | CM009691.2 | C2845_PM02G13780 | nonsynonymous SNP | A A | G G | la-related protein 1C |
| 8 | 2 | CM009691.2 | C2845_PM02G36960 | nonsynonymous SNP | C C | T T | protein RRP6-like 1 isoform X2 |
| 9 | 2 | CM009691.2 | C2845_PM02G37450 | nonsynonymous SNP | T T | C C | zinc finger protein GLI1-like isoform X2 |
| 10 | 2 | CM009691.2 | C2845_PM02G37550 | nonsynonymous SNP | T T | C C | protein SHOOT GRAVITROPISM 6 |
| 11 | 2 | CM009691.2 | C2845_PM02G37680 | nonsynonymous SNP | C C | T T | beta-glucuronosyltransferase GlcAT14A |
| 12 | 2 | CM009691.2 | C2845_PM02G37710 | nonsynonymous SNP | T T | A A | LOW QUALITY PROTEIN: probable F-box protein At4g22165 |
| 13 | 2 | CM009691.2 | C2845_PM02G37840 | nonsynonymous SNP | C C | T T | chloroplastic group IIA intron splicing facilitator CRS1, chloroplastic-like (LOC120681600), transcript variant X36, misc_RNA |
| 14 | 3 | CM009692.2 | C2845_PM03G07460 | nonsynonymous SNP | T T | C C | protein trichome birefringence-like 13 |
| 15 | 3 | CM009692.2 | C2845_PM03G10080 | nonsynonymous SNP | C C | A A | uncharacterized protein |
| 16 | 3 | CM009692.2 | C2845_PM03G10080 | nonsynonymous SNP | G G | A A | uncharacterized protein |
| 17 | 3 | CM009692.2 | C2845_PM03G21350 | nonsynonymous SNP | G G | A A | nucleotide excision repair protein homolog |
| 18 | 3 | CM009692.2 | C2845_PM03G29880 | nonsynonymous SNP | A A | T T | G-type lectin S-receptor-like serine/threonine-protein kinase At1g11303 |
| 19 | 3 | CM009692.2 | C2845_PM03G29880 | nonsynonymous SNP | T T | G G | G-type lectin S-receptor-like serine/threonine-protein kinase At1g11303 |
| 20 | 3 | CM009692.2 | C2845_PM03G29880 | nonsynonymous SNP | A A | G G | G-type lectin S-receptor-like serine/threonine-protein kinase At1g11303 |
| 21 | 4 | CM009693.2 | C2845_PM04G03050 | nonsynonymous SNP | A A | C C | small RNA 2 |
| 22 | 4 | CM009693.2 | C2845_PM04G04350 | nonsynonymous SNP | A A | C C | uncharacterized protein |
| 23 | 4 | CM009693.2 | C2845_PM04G04350 | nonsynonymous SNP | C C | T T | uncharacterized protein |
| 24 | 4 | CM009693.2 | C2845_PM04G26330 | nonsynonymous SNP | C C | T T | serine/arginine repetitive matrix protein 1-like isoform X1 |
| **Sl. No.** | **Chromosome number** | **GenBank accession** | **Gene Locus** | **SNP type** | **Wild type** | **High yielding mutant** | **Gene Information** |
| 25 | 5 | CM009694.2 | C2845_PM05G13140 | nonsynonymous SNP | G G | A A | phenylalanine--tRNA ligase beta subunit, cytoplasmic |
| 26 | 5 | CM009694.2 | C2845_PM05G15150 | nonsynonymous SNP | A A | G G | ABC transporter G family member 29-like |
| 27 | 5 | CM009694.2 | C2845_PM05G16740 | nonsynonymous SNP | G G | A A | uncharacterized protein |
| 28 | 5 | CM009694.2 | C2845_PM05G17020 | nonsynonymous SNP | A A | T T | ureide permease 4-like |
| 29 | 5 | CM009694.2 | C2845_PM05G25760 | nonsynonymous SNP | G G | A A | MAR-binding filament-like protein 1 |
| 30 | 6 | CM009695.2 | C2845_PM06G03480 | nonsynonymous SNP | T T | A A | paired amphipathic helix protein Sin3-like 3 |
| 31 | 6 | CM009695.2 | C2845_PM06G11750 | nonsynonymous SNP | G G | A A | filament-like plant protein 1 |
| 32 | 6 | CM009695.2 | C2845_PM06G11880 | nonsynonymous SNP | A A | G G | putative CCA tRNA nucleotidyltransferase 2 |
| 33 | 6 | CM009695.2 | C2845_PM06G13130 | nonsynonymous SNP | G G | T T | NLR family CARD domain-containing protein 3 |
| 34 | 6 | CM009695.2 | C2845_PM06G14390 | nonsynonymous SNP | G G | C C | uncharacterized protein |
| 35 | 6 | CM009695.2 | C2845_PM06G25390 | nonsynonymous SNP | T T | G G | ATP-dependent DNA helicase PIF2-like |
| 36 | 6 | CM009695.2 | C2845_PM06G28790 | stopgain SNP | T T | G G | replication protein A 70 kDa DNA-binding subunit B-like |
| 37 | 6 | CM009695.2 | C2845_PM06G35540 | nonsynonymous SNP | T T | G G | carotenoid 9,10 |
| 38 | 7 | CM009696.2 | C2845_PM07G13440 | nonsynonymous SNP | T T | C C | hypothetical protein |
| 39 | 7 | CM009696.2 | C2845_PM07G25570 | nonsynonymous SNP | C C | T T | 60S ribosomal protein L24 |
| 40 | 7 | CM009696.2 | C2845_PM07G28530 | nonsynonymous SNP | T T | C C | aldose reductase |
| 41 | 8 | CM009697.2 | C2845_PM08G16750 | nonsynonymous SNP | A A | G G | hypothetical protein |
| 42 | 8 | CM009697.2 | C2845_PM08G16750 | nonsynonymous SNP | C C | G G | hypothetical protein |
| 43 | 8 | CM009697.2 | C2845_PM08G16750 | nonsynonymous SNP | T T | C C | hypothetical protein |
| 44 | 8 | CM009697.2 | C2845_PM08G16750 | stoploss SNP | T T | C C | hypothetical protein |
| 45 | 11 | CM009700.2 | C2845_PM11G15190 | nonsynonymous SNP | A A | G G | BEACH domain-containing protein B |
| 46 | 11 | CM009700.2 | C2845_PM11G22260 | nonsynonymous SNP | T T | C C | putative F-box protein PP2-B12 |
| 47 | 12 | CM009701.2 | C2845_PM12G14980 | nonsynonymous SNP | T T | C C | glucan endo-1,3-beta-glucosidase-like protein 3 |
| 48 | 12 | CM009701.2 | C2845_PM12G26030 | nonsynonymous SNP | G G | A A | NEP1-interacting protein-like 2 |
| 49 | 12 | CM009701.2 | C2845_PM12G26300 | nonsynonymous SNP | A A | G G | dynamin-2A-like |
| 50 | 12 | CM009701.2 | C2845_PM12G29980 | nonsynonymous SNP | A A | T T | P-loop guanosine triphosphatase YjiA-like |
| 51 | 12 | CM009701.2 | C2845_PM12G31080 | nonsynonymous SNP | A A | G G | pumilio homolog 1-like |
| 52 | 13 | CM009702.2 | C2845_PM13G01550 | nonsynonymous SNP | A A | G G | hypothetical protein |
| 53 | 13 | CM009702.2 | C2845_PM13G02170 | nonsynonymous SNP | T T | C C | putative beta-1,3-galactosyltransferase 20 |
| **Sl. No.** | **Chromosome number** | **GenBank accession** | **Gene Locus** | **SNP type** | **Wild type** | **High yielding mutant** | **Gene Information** |
| 54 | 13 | CM009702.2 | C2845_PM13G02310 | nonsynonymous SNP | A A | T T | putative protein kinase gene, partial cds; putative Cf-2, fertilization-independent endosperm proteins, hypothetical protein, putative non-LTR retroelement reverse transcriptase, OCL5 protein, tryptophan synthase beta-subunit, hypothetical proteins, putative AP endonuclease, putative RNA polymerase II complex component SRB7, putative beta-1,3-glucanase, hypothetical protein, TNP2-like protein, hypothetical protein, putative phosphate/phosphoenolpyruvate translocator, putative protein, hypothetical proteins, putative galactosyltransferase family, hypothetical protein, putative cytochrome P450 family, putative lipid transfer protein, putative photoreceptor-interacting protein, and hypothetical protein genes, complete cds; and hypothetical protein gene, partial cds |
| 55 | 13 | CM009702.2 | C2845_PM13G08080 | nonsynonymous SNP | A A | G G | aspartate carbamoyltransferase 1 |
| 56 | 13 | CM009702.2 | C2845_PM13G11460 | nonsynonymous SNP | T T | C C | nuclear intron maturase 1, mitochondrial-like |
| 57 | 13 | CM009702.2 | C2845_PM13G11460 | nonsynonymous SNP | T T | C C | hypothetical protein |
| 58 | 13 | CM009702.2 | C2845_PM13G11460 | nonsynonymous SNP | C C | G G | hypothetical protein |
| 59 | 13 | CM009702.2 | C2845_PM13G11930 | nonsynonymous SNP | A A | G G | hydroxyproline-rich glycoprotein-like |
| 60 | 13 | CM009702.2 | C2845_PM13G11930 | nonsynonymous SNP | C C | T T | hydroxyproline-rich glycoprotein-like |
| 61 | 13 | CM009702.2 | C2845_PM13G13030 | nonsynonymous SNP | T T | C C | ferrochelatase-1, chloroplastic |
| 62 | 13 | CM009702.2 | C2845_PM13G13050 | nonsynonymous SNP | C C | A A | putative 1-phosphatidylinositol-3-phosphate 5-kinase FAB1C |
| 63 | 13 | CM009702.2 | C2845_PM13G18940 | nonsynonymous SNP | T T | C C | 1-phosphatidylinositol-3-phosphate 5-kinase FAB1B-like |
| 64 | 13 | CM009702.2 | C2845_PM13G19510 | nonsynonymous SNP | G G | A A | activator-like transposable element |
| 65 | 14 | CM009703.2 | C2845_PM14G17030 | nonsynonymous SNP | C C | T T | proline-rich protein 36-like |
| 66 | 14 | CM009703.2 | C2845_PM14G17320 | nonsynonymous SNP | C C | G G | tRNA (cytosine(34)-C (5))-methyltransferase-like |
| 67 | 14 | CM009703.2 | C2845_PM14G19120 | nonsynonymous SNP | A A | G G | MADS-box transcription factor 56-like |
| 68 | 14 | CM009703.2 | C2845_PM14G21220 | nonsynonymous SNP | A A | G G | transmembrane 9 superfamily member 8 |
| **Sl. No.** | **Chromosome number** | **GenBank accession** | **Gene Locus** | **SNP type** | **Wild type** | **High yielding mutant** | **Gene Information** |
| 69 | 15 | CM009704.2 | C2845_PM15G03350 | nonsynonymous SNP | T T | C C | probable ribonuclease P/MRP protein subunit POP5 |
| 70 | 15 | CM009704.2 | C2845_PM15G08140 | nonsynonymous SNP | G G | A A | ATP-dependent DNA helicase PIF1-like |
| 71 | 15 | CM009704.2 | C2845_PM15G08140 | nonsynonymous SNP | G G | A A | ATP-dependent DNA helicase PIF1-like |
| 72 | 15 | CM009704.2 | C2845_PM15G08310 | nonsynonymous SNP | G G | A A | ubiquitin carboxyl-terminal hydrolase 27-like |
| 73 | 15 | CM009704.2 | C2845_PM15G08310 | nonsynonymous SNP | T T | C C | hypothetical protein |
| 74 | 15 | CM009704.2 | C2845_PM15G26970 | nonsynonymous SNP | C C | G G | farnesylcysteine lyase |
| 75 | 16 | CM009705.2 | C2845_PM16G04220 | nonsynonymous SNP | A A | T T | putative protein phosphatase 2C 38 |
| 76 | 16 | CM009705.2 | C2845_PM16G04520 | nonsynonymous SNP | T T | A A | putative B3 domain-containing protein Os04g0347400 |
| 77 | 16 | CM009705.2 | C2845_PM16G04520 | nonsynonymous SNP | A A | G G | hypothetical protein |
| 78 | 16 | CM009705.2 | C2845_PM16G05820 | nonsynonymous SNP | G G | A A | calmodulin-binding transcription activator 4 isoform X1 |
| 79 | 17 | CM009706.2 | C2845_PM17G03890 | nonsynonymous SNP | C C | A A | DNA polymerase delta catalytic subunit |
| 80 | 17 | CM009706.2 | C2845_PM17G11490 | nonsynonymous SNP | C C | A A | serine/threonine-protein kinase-like protein |
| 81 | 17 | CM009706.2 | C2845_PM17G13390 | nonsynonymous SNP | A A | G G | monosaccharide-sensing protein 3-like |
| 82 | 18 | CM009707.2 | C2845_PM18G00870 | stopgain SNP | A A | C C | rho GTPase-activating protein 4-like |
| 83 | 18 | CM009707.2 | C2845_PM18G12810 | nonsynonymous SNP | G G | A A | serine/arginine-rich SC35-like splicing factor SCL30 |
| 84 | NA | PQIB02000765.1 | C2845_PMPSC055818 | nonsynonymous SNP | T T | G G | type I inositol polyphosphate 5-phosphatase 10-like |

**S 5. Details of functional single nucleotide polymorphisms (SNPs) identified between wild type and low yielding mutant**

| **Sl. No.** | **Chromosome number** | **GenBank accession** | **Gene Locus** | **SNP type** | **Wild type** | **Low yielding mutant** | **Gene Information** |
| --- | --- | --- | --- | --- | --- | --- | --- |
| 1 | 1 | CM009690.2 | C2845_PM01G00560 | nonsynonymous SNP | A A | G G | deSI-like protein |
| 2 | 1 | CM009690.2 | C2845_PM01G09740 | nonsynonymous SNP | A A | G G | imidazole glycerol phosphate synthase hisHF, chloroplastic |
| 3 | 1 | CM009690.2 | C2845_PM01G19760 | nonsynonymous SNP | T T | C C | lysine-specific histone demethylase 1 homolog 3 |
| 4 | 1 | CM009690.2 | "C2845_PM01G28280" | nonsynonymous SNP | T T | G G | uncharacterized protein |
| 5 | 1 | CM009690.2 | C2845_PM01G28600 | nonsynonymous SNP | A A | G G | E3 ubiquitin protein ligase DRIP2-like |
| 6 | 1 | CM009690.2 | C2845_PM01G28680 | nonsynonymous SNP | C C | T T | CWF19-like protein 2 |
| 7 | 1 | CM009690.2 | C2845_PM01G28680 | nonsynonymous SNP | C C | T T | same as above |
| 8 | 1 | CM009690.2 | C2845_PM01G33970 | nonsynonymous SNP | C C | T T | DNA-directed RNA polymerase II subunit RPB2 |
| 9 | 1 | CM009690.2 | C2845_PM01G33980 | nonsynonymous SNP | A A | C C | translation initiation factor eIF-2B subunit delta isoform X1 |
| 10 | 1 | CM009690.2 | C2845_PM01G34580 | nonsynonymous SNP | C C | A A | protein NRT1/ PTR FAMILY 1.2-like |
| 11 | 1 | CM009690.2 | C2845_PM01G37690 | nonsynonymous SNP | G G | A A | protein Rf1, mitochondrial-like |
| 12 | 2 | CM009691.2 | C2845_PM02G02450 | nonsynonymous SNP | A A | G G | E3 ubiquitin-protein ligase UPL7 |
| 13 | 3 | CM009692.2 | C2845_PM03G16590 | nonsynonymous SNP | T T | C C | ricin B-like lectin R40G3, clone 265197 stress responsive protein mRNA |
| 14 | 3 | CM009692.2 | C2845_PM03G16990 | nonsynonymous SNP | A A | C C | serine/threonine-protein kinase TIO-like isoform X2 |
| 15 | 3 | CM009692.2 | C2845_PM03G27270 | stopgain SNP | C C | T T | putative pentatricopeptide repeat-containing protein At5g08310, |
| 16 | 3 | CM009692.2 | C2845_PM01G42790 | nonsynonymous SNP | T T | C C | ent-copalyl diphosphate synthase 1, chloroplastic-like isoform X2 |

| **Sl. No.** | **Chromosome number** | **GenBank accession** | **Gene Locus** | **SNP type** | **Wild type** | **Low yielding mutant** | **Gene Information** |
| --- | --- | --- | --- | --- | --- | --- | --- |
| 17 | 3 | CM009692.2 | C2845_PM03G29880 | nonsynonymous SNP | A A | T T | G-type lectin S-receptor-like serine/threonine-protein kinase At1g11303 |
| 18 | 3 | CM009692.2 | C2845_PM03G29880 | nonsynonymous SNP | T T | G G | G-type lectin S-receptor-like serine/threonine-protein kinase At1g11303 |
| 19 | 3 | CM009692.2 | C2845_PM03G29880 | nonsynonymous SNP | A A | G G | G-type lectin S-receptor-like serine/threonine-protein kinase At1g11303 |
| 20 | 3 | CM009692.2 | C2845_PM03G30800 | nonsynonymous SNP | T T | C C | ferrochelatase-1, chloroplastic |
| 21 | 4 | CM009693.2 | C2845_PM04G03050 | nonsynonymous SNP | A A | C C | small RNA 2 |
| 22 | 4 | CM009693.2 | C2845_PM04G04350 | nonsynonymous SNP | A A | C C | uncharacterized protein |
| 23 | 5 | CM009694.2 | C2845_PM05G01420 | nonsynonymous SNP | A A | G G | GTPase LSG1-2-like |
| 24 | 5 | CM009694.2 | C2845_PM05G01910 | nonsynonymous SNP | A A | G G | hypothetical protein |
| 25 | 5 | CM009694.2 | C2845_PM05G05260 | nonsynonymous SNP | C C | T T | ribonuclease H2 subunit A-like |
| 26 | 5 | CM009694.2 | C2845_PM05G08510 | nonsynonymous SNP | A A | C C | Peroxidase,alpha-1,2-Mannosidase, UPI0001A840FE related cluster,Nucleobase-ascorbate transporter 7 |
| 27 | 5 | CM009694.2 | C2845_PM05G10250 | nonsynonymous SNP | T T | A A | replication protein A 70 kDa DNA-binding subunit C-like |
| 28 | 5 | CM009694.2 | C2845_PM05G15150 | nonsynonymous SNP | A A | G G | ABC transporter G family member 29-like |
| 29 | 5 | CM009694.2 | C2845_PM05G17020 | nonsynonymous SNP | A A | T T | ureide permease 4-like |
| 30 | 5 | CM009694.2 | C2845_PM05G17900 | nonsynonymous SNP | G G | C C | hypothetical protein |
| 31 | 5 | CM009694.2 | C2845_PM05G18190 | nonsynonymous SNP | T T | G G | hypothetical protein |
| 32 | 5 | CM009694.2 | C2845_PM05G18700 | nonsynonymous SNP | C C | G G | DExH-box ATP-dependent RNA helicase DExH10-like |
| **Sl. No.** | **Chromosome number** | **GenBank accession** | **Gene Locus** | **SNP type** | **Wild type** | **Low yielding mutant** | **Gene Information** |
| 33 | 5 | CM009694.2 | C2845_PM05G19990 | nonsynonymous SNP | G G | A A | phosphoglucan, water dikinase, chloroplastic |
| 34 | 5 | CM009694.2 | C2845_PM05G22670 | nonsynonymous SNP | C C | T T | cation-chloride cotransporter 1 |
| 35 | 5 | CM009694.2 | C2845_PM05G23920 | nonsynonymous SNP | A A | G G | AP-3 complex subunit mu |
| 36 | 5 | CM009694.2 | C2845_PM05G28370 | nonsynonymous SNP | G G | A A | sister chromatid cohesion 1 protein 1 |
| 37 | 5 | CM009694.2 | C2845_PM05G31060 | nonsynonymous SNP | A A | T T | putative choline kinase 2 isoform X1 |
| 38 | 5 | CM009694.2 | C2845_PM05G31170 | nonsynonymous SNP | C C | T T | putative starch synthase 4,chloroplastic/amyloplastic |
| 39 | 5 | CM009694.2 | C2845_PM05G35150 | nonsynonymous SNP | C C | A A | selT-like protein |
| 40 | 6 | CM009695.2 | C2845_PM06G03480 | nonsynonymous SNP | T T | A A | paired amphipathic helix protein Sin3-like 3 |
| 41 | 6 | CM009695.2 | C2845_PM06G14390 | nonsynonymous SNP | G G | C C | uncharacterized protein |
| 42 | 6 | CM009695.2 | C2845_PM06G18240 | nonsynonymous SNP | T T | G G | uncharacterized protein |
| 43 | 6 | CM009695.2 | C2845_PM06G21550 | nonsynonymous SNP | C C | T T | C2845_PM06G21550 |
| 44 | 6 | CM009695.2 | C2845_PM06G22280 | nonsynonymous SNP | C C | T T | uncharacterized protein |
| 45 | 6 | CM009695.2 | C2845_PM06G22280 | nonsynonymous SNP | T T | G G | uncharacterized protein |
| 46 | 6 | CM009695.2 | C2845_PM06G22650 | nonsynonymous SNP | G G | A A | CSC1-like protein At4g02900, pentatricopeptide repeat-containing protein At1g09900-like |
| 47 | 6 | CM009695.2 | C2845_PM06G23850 | nonsynonymous SNP | G G | T T | transposon protein, putative, Mutator sub-class |
| 48 | 6 | CM009695.2 | C2845_PM06G24400 | nonsynonymous SNP | A A | C C | DNA primase small subunit |
| 49 | 6 | CM009695.2 | C2845_PM06G24630 | stoploss SNP | A A | G G | hypothetical protein |
| 50 | 6 | CM009695.2 | C2845_PM06G26530 | nonsynonymous SNP | T T | C C | putative protein kinase superfamily protein |
| **Sl. No.** | **Chromosome number** | **GenBank accession** | **Gene Locus** | **SNP type** | **Wild type** | **Low yielding mutant** | **Gene Information** |
| 51 | 7 | CM009696.2 | C2845_PM07G02740 | nonsynonymous SNP | A A | G G | nucleolin |
| 52 | 7 | CM009696.2 | C2845_PM07G13440 | nonsynonymous SNP | T T | C C | hypothetical protein |
| 53 | 7 | CM009696.2 | C2845_PM07G13740 | nonsynonymous SNP | G G | A A | serine/threonine-protein phosphatase 6 regulatory subunit 3-like isoform X2 |
| 54 | 7 | CM009696.2 | C2845_PM07G21730 | nonsynonymous SNP | G G | A A | hypothetical protein |
| 55 | 7 | CM009696.2 | C2845_PM07G23610 | nonsynonymous SNP | A A | C C | phosphoenolpyruvate carboxylase, housekeeping isozyme |
| 56 | 7 | CM009696.2 | C2845_PM07G35010 | nonsynonymous SNP | A A | T T | putative amidohydrolase YtcJ |
| 57 | 8 | CM009697.2 | C2845_PM08G04790 | nonsynonymous SNP | T T | C C | hypothetical protein |
| 58 | 8 | CM009697.2 | C2845_PM08G08930 | stopgain SNP | G G | A A | uncharacterized protein |
| 59 | 8 | CM009697.2 | C2845_PM08G13050 | nonsynonymous SNP | C C | T T | protein ECERIFERUM 3-like |
| 60 | 9 | CM009698.2 | C2845_PM09G01470 | stoploss SNP | T T | A A | protein TPX2 isoform X1 |
| 61 | 9 | CM009698.2 | C2845_PM09G02970 | nonsynonymous SNP | C C | T T | hypothetical protein |
| 62 | 9 | CM009698.2 | C2845_PM09G03030 | nonsynonymous SNP | T T | A A | hypothetical protein |
| 63 | 9 | CM009698.2 | C2845_PM09G07380 | nonsynonymous SNP | T T | C C | hypothetical protein |
| 64 | 9 | CM009698.2 | C2845_PM09G23290 | nonsynonymous SNP | T T | G G | AP-3 complex subunit mu |
| 65 | 10 | CM009699.2 | C2845_PM10G07330 | nonsynonymous SNP | A A | T T | receptor kinase-like protein Xa21 |
| 66 | 10 | CM009699.2 | C2845_PM10G07330 | nonsynonymous SNP | T T | G G | receptor kinase-like protein Xa21 |
| 67 | 10 | CM009699.2 | C2845_PM10G07600 | nonsynonymous SNP | A A | C C | vacuolar amino acid transporter 1-like |
| 68 | 10 | CM009699.2 | C2845_PM10G10130 | nonsynonymous SNP | T T | C C | putative membrane protein |
| 69 | 10 | CM009699.2 | C2845_PM10G16720 | nonsynonymous SNP | A A | C C | alpha/beta hydrolase domain-containing protein 17C-like |
| **Sl. No.** | **Chromosome number** | **GenBank accession** | **Gene Locus** | **SNP type** | **Wild type** | **Low yielding mutant** | **Gene Information** |
| 70 | 10 | CM009699.2 | C2845_PM10G17190 | nonsynonymous SNP | G G | T T | protein S-acyltransferase 24-like |
| 71 | 10 | CM009699.2 | C2845_PM10G19700 | nonsynonymous SNP | C C | T T | RNA polymerase I-specific transcription |
| 72 | 11 | CM009700.2 | C2845_PM11G08000 | nonsynonymous SNP | C C | T T | **auxin response factor 7** |
| 73 | 11 | CM009700.2 | C2845_PM11G09790 | nonsynonymous SNP | G G | A A | protein NBR1 homolog; protein JOKA2-like |
| 74 | 11 | CM009700.2 | C2845_PM11G14930 | nonsynonymous SNP | C C | G G | ATP-dependent RNA helicase SKI2 |
| 75 | 12 | CM009701.2 | C2845_PM12G06380 | nonsynonymous SNP | A A | G G | putative LRR receptor-like serine/threonine-protein kinase |
| 76 | 12 | CM009701.2 | C2845_PM12G06750 | nonsynonymous SNP | G G | A A | RING-H2 finger protein ATL46-like |
| 77 | 12 | CM009701.2 | C2845_PM12G08810 | nonsynonymous SNP | A A | C C | hypothetical protein |
| 78 | 12 | CM009701.2 | C2845_PM12G08820 | nonsynonymous SNP | A A | G G | BAG family molecular chaperone regulator 6 |
| 79 | 12 | CM009701.2 | C2845_PM12G08820 | nonsynonymous SNP | C C | T T | hypothetical protein |
| 80 | 12 | CM009701.2 | C2845_PM12G09590 | nonsynonymous SNP | C C | T T | hypothetical protein |
| 81 | 12 | CM009701.2 | C2845_PM12G13230 | nonsynonymous SNP | G G | T T | probable WRKY transcription factor 19; loricrin-related |
| 82 | 12 | CM009701.2 | C2845_PM12G14060 | nonsynonymous SNP | C C | T T | putative germin-like protein 2-2 |
| 83 | 12 | CM009701.2 | C2845_PM12G14110 | nonsynonymous SNP | T T | A A | putative germin-like protein 2-2 |
| 84 | 12 | CM009701.2 | C2845_PM12G14110 | nonsynonymous SNP | A A | C C | putative germin-like protein 2-2 |
| 85 | 12 | CM009701.2 | C2845_PM12G14110 | nonsynonymous SNP | A A | C C | putative germin-like protein 2-2 |
| 86 | 12 | CM009701.2 | C2845_PM12G14980 | nonsynonymous SNP | T T | C C | glucan endo-1,3-beta-glucosidase-like protein 3 |
| 87 | 12 | CM009701.2 | C2845_PM12G16290 | nonsynonymous SNP | T T | C C | protein PSK SIMULATOR 1-like |
| **Sl. No.** | **Chromosome number** | **GenBank accession** | **Gene Locus** | **SNP type** | **Wild type** | **Low yielding mutant** | **Gene Information** |
| 88 | 12 | CM009701.2 | C2845_PM12G17120 | nonsynonymous SNP | A A | G G | GPN-loop GTPase QQT1 |
| 89 | 12 | CM009701.2 | C2845_PM12G20330 | nonsynonymous SNP | T T | C C | calcium uptake protein, mitochondrial-like |
| 90 | 12 | CM009701.2 | C2845_PM12G21060 | nonsynonymous SNP | A A | G G | phytosulfokine receptor 1-like |
| 91 | 12 | CM009701.2 | C2845_PM12G26030 | nonsynonymous SNP | G G | A A | NEP1-interacting protein-like 2 |
| 92 | 13 | CM009702.2 | C2845_PM13G00630 | nonsynonymous SNP | A A | G G | uncharacterized protein |
| 93 | 13 | CM009702.2 | C2845_PM13G05300 | nonsynonymous SNP | C C | T T | starch synthase 3,chloroplastic/amyloplastic-like |
| 94 | 13 | CM009702.2 | C2845_PM13G06070 | nonsynonymous SNP | C C | T T | putative gamma-aminobutyrate transaminase 3,mitochondrial |
| 95 | 13 | CM009702.2 | C2845_PM13G07150 | nonsynonymous SNP | G G | A A | disease resistance protein RPM1-like |
| 96 | 13 | CM009702.2 | C2845_PM13G08080 | nonsynonymous SNP | A A | G G | aspartate carbamoyltransferase 1 |
| 97 | 13 | CM009702.2 | C2845_PM13G11460 | nonsynonymous SNP | T T | C C | nuclear intron maturase 1, mitochondrial-like; serine carboxypeptidase-like 34 |
| 98 | 13 | CM009702.2 | C2845_PM13G11460 | nonsynonymous SNP | T T | C C | nuclear intron maturase 1, mitochondrial-like; serine carboxypeptidase-like 34 |
| 99 | 13 | CM009702.2 | C2845_PM13G11460 | nonsynonymous SNP | C C | G G | nuclear intron maturase 1, mitochondrial-like; serine carboxypeptidase-like 34 |
| 100 | 13 | CM009702.2 | C2845_PM13G11930 | nonsynonymous SNP | A A | G G | hydroxyproline-rich glycoprotein-like |
| 101 | 13 | CM009702.2 | C2845_PM13G11930 | nonsynonymous SNP | C C | T T | hydroxyproline-rich glycoprotein-like |
| 102 | 13 | CM009702.2 | C2845_PM13G13030 | nonsynonymous SNP | T T | C C | ferrochelatase-1, chloroplastic |
| 103 | 13 | CM009702.2 | C2845_PM13G13050 | nonsynonymous SNP | C C | A A | putative 1-phosphatidylinositol-3-phosphate 5-kinase FAB1C |

| **Sl. No.** | **Chromosome number** | **GenBank accession** | **Gene Locus** | **SNP type** | **Wild type** | **Low yielding mutant** | **Gene Information** |
| --- | --- | --- | --- | --- | --- | --- | --- |
| 104 | 13 | CM009702.2 | C2845_PM13G14840 | stopgain SNP | T T | G G | 5'-3' exonuclease |
| 105 | 13 | CM009702.2 | C2845_PM13G15750 | nonsynonymous SNP | C C | T T | signal recognition particle subunit SRP72-like isoform X |
| 106 | 13 | CM009702.2 | C2845_PM13G18940 | nonsynonymous SNP | T T | C C | 1-phosphatidylinositol-3-phosphate 5-kinase FAB1B-like |
| 107 | 13 | CM009702.2 | C2845_PM13G19510 | nonsynonymous SNP | G G | A A | activator-like transposable element |
| 108 | 13 | CM009702.2 | C2845_PM13G20330 | nonsynonymous SNP | T T | C C | ubiquitin carboxyl-terminal hydrolase 16-like |
| 109 | 13 | CM009702.2 | C2845_PM13G21080 | nonsynonymous SNP | A A | G G | C2 and GRAM domain-containing protein |
| 110 | 13 | CM009702.2 | C2845_PM13G21170 | nonsynonymous SNP | G G | C C | putative galactinol--sucrose galactosyltransferase 1 |
| 111 | 13 | CM009702.2 | C2845_PM13G21240 | nonsynonymous SNP | A A | G G | phosphatidylinositol/phosphatidylcholine transfer protein SFH6-like |
| 112 | 13 | CM009702.2 | C2845_PM13G21800 | nonsynonymous SNP | G G | A A | glycerophosphodiester phosphodiesterase GDPDL4-like |
| 113 | 13 | CM009702.2 | C2845_PM13G22290 | nonsynonymous SNP | A A | G G | AT-hook motif nuclear-localized protein 10 |
| 114 | 13 | CM009702.2 | C2845_PM13G26450 | nonsynonymous SNP | C C | G G | structural maintenance of chromosomes protein 1 |
| 115 | 14 | CM009703.2 | C2845_PM14G05250 | nonsynonymous SNP | G G | A A | methionine aminopeptidase 1C,chloroplastic/mitochondrial-like isoform X1 |
| 116 | 14 | CM009703.2 | C2845_PM14G17030 | nonsynonymous SNP | C C | T T | proline-rich protein 36-like; nascent polypeptide-associated complex subunit alpha muscle-specific form-like; SH3 domain-containing protein C23A1.17-like; protein transport protein sec31-like |
| 117 | 14 | CM009703.2 | C2845_PM14G17320 | nonsynonymous SNP | C C | G G | tRNA (cytosine(34)-C(5))-methyltransferase-like |
| 118 | 14 | CM009703.2 | C2845_PM14G19120 | nonsynonymous SNP | A A | G G | MADS-box transcription factor 56-like |
| 119 | 14 | CM009703.2 | C2845_PM14G19120 | nonsynonymous SNP | G G | A A | MADS-box transcription factor 56-like |
| **Sl. No.** | **Chromosome number** | **GenBank accession** | **Gene Locus** | **SNP type** | **Wild type** | **Low yielding mutant** | **Gene Information** |
| 120 | 14 | CM009703.2 | C2845_PM14G21040 | nonsynonymous SNP | C C | T T | peptidyl-prolyl cis-trans isomerase CYP71 |
| 121 | 14 | CM009703.2 | C2845_PM14G21220 | nonsynonymous SNP | A A | G G | transmembrane 9 superfamily member 8 |
| 122 | 15 | CM009704.2 | C2845_PM15G03350 | nonsynonymous SNP | T T | C C | probable ribonuclease P/MRP protein subunit POP5 |
| 123 | 15 | CM009704.2 | C2845_PM15G03540 | nonsynonymous SNP | A A | T T | uncharacterized protein |
| 124 | 15 | CM009704.2 | C2845_PM15G04340 | nonsynonymous SNP | C C | T T | hypothetical protein |
| 125 | 15 | CM009704.2 | C2845_PM15G05620 | nonsynonymous SNP | A A | G G | L10-interacting MYB domain-containing protein-like |
| 126 | 15 | CM009704.2 | C2845_PM15G08140 | nonsynonymous SNP | G G | A A | ATP-dependent DNA helicase PIF1-like |
| 127 | 15 | CM009704.2 | C2845_PM15G08310 | nonsynonymous SNP | G G | A A | ubiquitin carboxyl-terminal hydrolase 27-like |
| 128 | 15 | CM009704.2 | C2845_PM15G08310 | nonsynonymous SNP | T T | C C | ubiquitin carboxyl-terminal hydrolase 27-like |
| 129 | 15 | CM009704.2 | C2845_PM15G11680 | nonsynonymous SNP | G G | A A | protein THYLAKOID ASSEMBLY 8-like |
| 130 | 15 | CM009704.2 | C2845_PM15G11720 | nonsynonymous SNP | C C | T T | pentatricopeptide repeat-containing protein At5g11310 |
| 131 | 15 | CM009704.2 | C2845_PM15G12150 | nonsynonymous SNP | T T | A A | mitochondrial inner membrane protease ATP23 |
| 132 | 15 | CM009704.2 | C2845_PM01G34750 | nonsynonymous SNP | T T | C C | B3 domain-containing protein Os03g0620400-like |
| 133 | 15 | CM009704.2 | C2845_PM15G14160 | nonsynonymous SNP | T T | A A | hypothetical protein |
| 134 | 15 | CM009704.2 | C2845_PM15G16200 | nonsynonymous SNP | A A | C C | subtilisin-like protease SBT2.5 |
| 135 | 15 | CM009704.2 | C2845_PM15G16200 | nonsynonymous SNP | A A | T T | subtilisin-like protease SBT2.5 |
| 136 | 15 | CM009704.2 | C2845_PM15G20870 | nonsynonymous SNP | G G | T T | V-type proton ATPase subunit G1-like |
| 137 | 15 | CM009704.2 | C2845_PM15G21950 | nonsynonymous SNP | C C | T T | myosin heavy chain, skeletal muscle |
| **Sl. No.** | **Chromosome number** | **GenBank accession** | **Gene Locus** | **SNP type** | **Wild type** | **Low yielding mutant** | **Gene Information** |
| 138 | 15 | CM009704.2 | C2845_PM15G22130 | nonsynonymous SNP | G G | T T | putative FBD-associated F-box protein At3g50710; flotillin-like protein 2 |
| 139 | 15 | CM009704.2 | C2845_PM15G22630 | nonsynonymous SNP | T T | C C | uncharacterized protein |
| 140 | 16 | CM009705.2 | C2845_PM16G02380 | nonsynonymous SNP | T T | C C | hypothetical protein |
| 141 | 16 | CM009705.2 | C2845_PM16G15220 | nonsynonymous SNP | T T | C C | SH3 domain-containing protein 2-like |
| 142 | 16 | CM009705.2 | C2845_PM16G16720 | nonsynonymous SNP | T T | C C | ubiquitin fusion degradation protein 1 homolog; heterogeneous nuclear ribonucleoprotein Q-like |
| 143 | 16 | CM009705.2 | C2845_PM16G17300 | nonsynonymous SNP | T T | C C | transport and Golgi organization 2 homolog |
| 144 | 16 | CM009705.2 | C2845_PM16G18520 | nonsynonymous SNP | G G | A A | E3 ubiquitin-protein ligase RNF14-like |
| 145 | 16 | CM009705.2 | C2845_PM16G22710 | nonsynonymous SNP | C C | T T | cupin gene, partial cds; and plastid glutamine synthetase 2 (GS2) gene, GS2-D1b allele |
| 146 | 16 | CM009705.2 | C2845_PM16G22880 | nonsynonymous SNP | T T | C C | serine/arginine repetitive matrix protein 2 isoform X2 |
| 147 | 16 | CM009705.2 | C2845_PM16G22880 | nonsynonymous SNP | T T | G G | serine/arginine repetitive matrix protein 2 isoform X2 |
| 148 | 16 | CM009705.2 | C2845_PM16G23070 | nonsynonymous SNP | A A | T T | peptidyl-prolyl cis-trans isomerase Pin1-like |
| 149 | 17 | CM009706.2 | C2845_PM17G04750 | nonsynonymous SNP | T T | G G | protein ACCELERATED CELL DEATH 6-like isoform X1 |
| 150 | 17 | CM009706.2 | C2845_PM17G05040 | nonsynonymous SNP | G G | A A | hypothetical protein |
| 151 | 17 | CM009706.2 | C2845_PM17G09340 | nonsynonymous SNP | A A | G G | Reverse transcriptase (RNA-dependent DNA polymerase), putative |
| 152 | 17 | CM009706.2 | C2845_PM17G11620 | nonsynonymous SNP | A A | G G | nucleolin |
| 153 | 17 | CM009706.2 | C2845_PM17G11670 | nonsynonymous SNP | G G | C C | sialyltransferase-like protein 5 |
| **Sl. No.** | **Chromosome number** | **GenBank accession** | **Gene Locus** | **SNP type** | **Wild type** | **Low yielding mutant** | **Gene Information** |
| 154 | 17 | CM009706.2 | C2845_PM17G13390 | nonsynonymous SNP | A A | G G | monosaccharide-sensing protein 3-like |
| 155 | 17 | CM009706.2 | C2845_PM17G14970 | nonsynonymous SNP | C C | T T | uncharacterized protein |
| 156 | 18 | CM009707.2 | C2845_PM18G00870 | stopgain SNP | A A | C C | rho GTPase-activating protein 4-like |
| 157 | 18 | CM009707.2 | C2845_PM18G03240 | nonsynonymous SNP | G G | A A | hypothetical protein |
| 158 | 18 | CM009707.2 | C2845_PM18G03550 | nonsynonymous SNP | G G | A A | MATH domain-containing protein |
| 159 | 18 | CM009707.2 | C2845_PM18G04240 | nonsynonymous SNP | T T | C C | putative disease resistance protein RGA1, cysteine-rich receptor-like protein kinase 5 |
| 160 | 18 | CM009707.2 | C2845_PM18G05400 | nonsynonymous SNP | C C | A A | phosphoinositide phosphatase SAC7-like |
| 161 | 18 | CM009707.2 | C2845_PM18G05400 | nonsynonymous SNP | A A | G G | phosphoinositide phosphatase SAC7-like |
| 162 | 18 | CM009707.2 | C2845_PM18G05540 | stopgain SNP | T T | C C | plant UBX domain-containing protein 11 |
| 163 | 18 | CM009707.2 | C2845_PM18G05600 | nonsynonymous SNP | T T | C C | putative gag-pol polyprotein |
| 164 | 18 | CM009707.2 | C2845_PM18G05600 | nonsynonymous SNP | A A | G G | putative gag-pol polyprotein |
| 165 | 18 | CM009707.2 | C2845_PM18G05600 | nonsynonymous SNP | A A | C C | putative gag-pol polyprotein |
| 166 | 18 | CM009707.2 | C2845_PM18G06300 | nonsynonymous SNP | G G | A A | uncharacterized protein |
| 167 | 18 | CM009707.2 | C2845_PM18G07130 | nonsynonymous SNP | G G | C C | putative disease resistance RPP13-like protein 1 |
| 168 | 18 | CM009707.2 | C2845_PM18G08150 | nonsynonymous SNP | G G | A A | hypothetical protein |
| 169 | 18 | CM009707.2 | C2845_PM18G12060 | nonsynonymous SNP | T T | A A | nodulin homeobox; sodium/hydrogen exchanger 1-like |
| 170 | 18 | CM009707.2 | C2845_PM18G12810 | nonsynonymous SNP | G G | A A | serine/arginine-rich SC35-like splicing factor SCL30 |
| 171 | NA | PQIB02000373.1 | C2845_PMPSC038591 | nonsynonymous SNP | C C | T T | hypothetical protein |
